# Polymer-Based Contactless Conductivity Detector for Europan Salts (PolyCoDES)

**DOI:** 10.3390/s25030775

**Published:** 2025-01-27

**Authors:** Chinmayee Govinda Raj, Peyton Salyards, Catherine McCoy, Amanda Stockton

**Affiliations:** 1 School of Chemistry and Biochemistry, Georgia Institute of Technology, 901 Atlantic Drive, Atlanta, GA 30332, USA; 2 Wheeler High School, Marietta, GA 30068, USA

**Keywords:** polymer electrodes, contactless conductivity detection, Europa, *in situ* analysis, planetary missions

## Abstract

This study presents the development of an innovative drop-stain-coat fabrication technique for creating high-quality PEDOT:PSS films, optimized for use in polymer-based electrodes within contactless conductivity detection (C^4^D) sensors. We detail the fabrication and thorough characterization of PEDOT films produced via the drop-stain-coat method, emphasizing its efficiency and reliability in electrode manufacturing. The resulting polymer electrodes were integrated into C^4^D sensors, which were rigorously characterized to assess their performance in detecting multiple salt types within real-world samples. This approach highlights the potential of drop-stain-coat fabrication to advance sensor applications in diverse analytical environments, offering a practical solution for accurate and adaptable conductivity detection.

## 1. Introduction

Since time immemorial, humans have pondered, “are we alone?’’. We have continually attempted to assess heavenly bodies for their potential to host life, with our search guided by the principle that we can recognize life as we know it. Mars, Europa, and Enceladus are established candidates for the search for life. Measuring a sample’s ionic content is a critical preliminary measurement to gain understanding of chemistry and mineralogy for habitability assessments. Mars’ regolith is believed to have sulfates and chlorides [1]. Although they seem to have formed on the surface during climatic processes [2], many appear to have been exhumed from the subsurface [3,4], needing subsurface analyses on a global scale to identify the origin. On Enceladus, carbonates have been detected [5], and Europa’s surface is rich in sulfates and chlorides [6,7]. However, space weathering products may hide the native composition below a layer of exogenic products on these bodies [8]. Data about subsurface ionic constitution from a wide geographical area are necessary. *In situ* analyses on these bodies are required to access samples protected from global-scale climatic processes, radiation, and space-weathering byproducts.

On Earth, capacitively coupled contactless detectors (C^4^D) have played a dominant role in electrochemical detection of ionic species on microfluidic platforms [9]. The earliest record of the possibility of conductance measurements with samples isolated from sensing electrodes are from 1928 [10]. The first capacitively coupled contactless conductivity detector (C^4^D) designs had two outer cylindrical electrodes aligned along the axis of a glass tube [9]. With the advent of microfluidics in the 1990s, the design has evolved to include small electrode tubes encompassing fused silica capillaries, effectively demonstrating C^4^D’s ability to function successfully as a microsensor [9,11]. Devices includes an electrode pair underneath a microchannel sandwiching an insulating layer. The electrodes are analogous to the two plates of a capacitor with microchannel contents as the dielectric medium—ionic concentration in the medium dominates the capacitive coupling of electrodes [11]. This architecture enables low possibility of electrode fouling, resulting in excellent repeatability. Microfluidic C^4^D devices are used frequently in point-of-care medical diagnostics [12], therapeutic monitoring [13], pharmacology [14], biomolecular analysis [15], environmental monitoring [16], and agricultural and food analysis [17,18], and have been proposed for space mission applications [19,20,21].

C^4^D microdevice fabrication is a three-step process and includes the fabrication of a microchannel, an insulating layer, and an electrode pair. Typical microchannel photolithography and glass microfabrication protocols demand weeks of time by trained personnel in a cleanroom facility, making development resource-intensive and costly. These fabrication procedures have matured from glass chips to polymeric materials due to lower cost and faster turn-around times [22,23,24], but still use SU-8 molds for microchannels, requiring cleanroom facilities for photolithography and wet-etching.

Spin-coating is typically used for fabricating the insulating film in the tens-of-micrometers thickness range [22,25,26], but suffers from low material efficiency and optimization of multiple parameters to maintain reproducibility [27,28]. A simpler, resource-efficient technique could enable structured, iterative prototyping cycles at a fraction of material and infrastructure cost.

Almost all C^4^D devices have used thin metallic electrodes like copper [29], platinum [30], silver [31], and aluminum [32,33]. Terrestrial applications, like rugged field portable diagnostic tools, use rigid and brittle materials. Flexibility and impact resistance can enable flexible, real-time wearable sensors on Earth, and open extraterrestrial missions like kinetic penetrator platforms that endure stresses in the 100s of MPa range from above 5 km/s landing speeds. The need for electrode materials offering higher potential for impact resistance is evident from these reports [34].

Flexible polymers like poly (3,4-ethylenedioxythiophene) polystyrene sulfonate (PEDOT:PSS) have modifiable electrical properties that are altered by changing their chemical structure using a variety of treatments including thermal [35,36], radiation [36,37], organic solvent [36,37], ionic solution [36,38], and acid treatment [36,39]. Sulfuric acid treatment yields the highest reported electrical conductivity [37,38,39,40,41], 4.38 × 10^3^ S/cm [42], resulting in wearable devices with stretchability/pliability [38], and in mechanically robust solar cells [43].

Electrode film fabrication techniques like spin-coating, dip-coating, spray-coating, and printing are widely used for generating thin polymer films. However, they come with their own limitations, which necessitate the development of a new technique. Issues with spin-coating has already been mentioned above. Dip-coating requires iterative coating steps based on the desired thickness [44], has adhering issues [45], could result in gravity-induced drainage during coating resulting in an uneven thickness [45,46], is not suited for selective are application [47], and can be resource-intensive [44]. Spray-coating, another well-used technique, has reproducibility issues [48,49], requires iterative coating steps [47] making it a time-consuming process, only to yield unpredictable deposit quality despite identical coating setups [48], and has adhering issues [49], while generally being unsuited for small area application. Lastly, printing also has issues with substrate quality’s heavy reliance on print speed and offers limited substrate choice [50]. Some groups have even reported requiring additives for uniform thickness and non-clustered polymer distribution, potentially decreasing the film conductance [51].

Due to the potential robustness of flexible polymer electrodes to high-g space mission accelerations, we use them to develop the Polymer-based Conductivity Detector for Europan Salts (PolyCoDES), a science payload capable of detecting low concentration salts on a penetrator mission. PolyCoDES uses a complete polymer architecture without cleanroom facilities. The microfabrication technique demonstrated by Morbioli et al. [52], and Speller et al. [53], enables a novel drop-stain-coat fabrication method for PEDOT:PSS layers as C^4^D electrodes. The technique is simple, resource-efficient, and enables iterative prototyping cycles with high reproducibility and short turnaround times. These characteristics make technology transfer for flight applications undemanding, while enabling microfluidic research and fabrication in low-resource areas of the world. PolyCoDES is a science payload capable of detecting low concentration salts on penetrator modules. Three devices are fabricated and tested with Europa-relevant salts (NaCl, MgSO_4_, Na_2_SO_4_, and KCl) and validated with two “real-world” samples (ocean water and water from Rio Tinto, Spain). This is the first report of the drop-stain-coating technique, and to the best of our knowledge, the first demonstration of a polymer C^4^D electrode.

The primary goal of this work Is to discuss the novel polymer electrode fabrication technique and its optimization; this is followed by device testing and performance validation. The fabrication method of the microchannel has been borrowed by Morbioli et al. [52] and Speller et al. [53] and integrated to with the polymer electrodes to build the PolyCODES device.

## 2. Materials and Methods

### 2.1. Reagents and Polymer Solution Preparation

Millipore water was filtered through three stages to reach 18 MΩ/cm resistivity and used to prepare all aqueous stock samples. Salt stock solutions were stored at 4 °C. Copper etching solution was made using 45 mL DI water, 10 mL 6 N hydrochloric acid (VWR, Solon, OH, USA), and 5 mL 30% hydrogen peroxide (Sigma Aldrich, St. Louis, MO, USA). Sodium chloride, magnesium sulfate, potassium chloride, and sodium sulfate were used as received (VWR, Solon, OH, USA). About 15 mL of SYLGARD 184 (Dow Corning, Midland, MI, USA) was prepared by mixing 10 parts elastomer base and 1 part curing agent by volume in 50 mL Falcon conical centrifuge tubes (Fisher Scientific, Waltham, MA, USA) and degassed to remove bubbles (~40 min). Conductive grade PEDOT:PSS (Sigma Aldrich, St. Louis, MO, USA) was filtered using 0.45 µm syringe filters (VWR, Solon, OH, USA) attached to 1 mL plastic syringes with luer lock connectors (VWR, Solon, OH, USA). PEDOT:PSS solutions were stored at 4 °C when not in use. Diluting 97% stock (Sigma Aldrich, St. Louis, MO, USA) yielded 6 M sulfuric acid for polymer film treatment.

### 2.2. PEDOT:PSS Film and Electrode Fabrication

**Figure 1:**
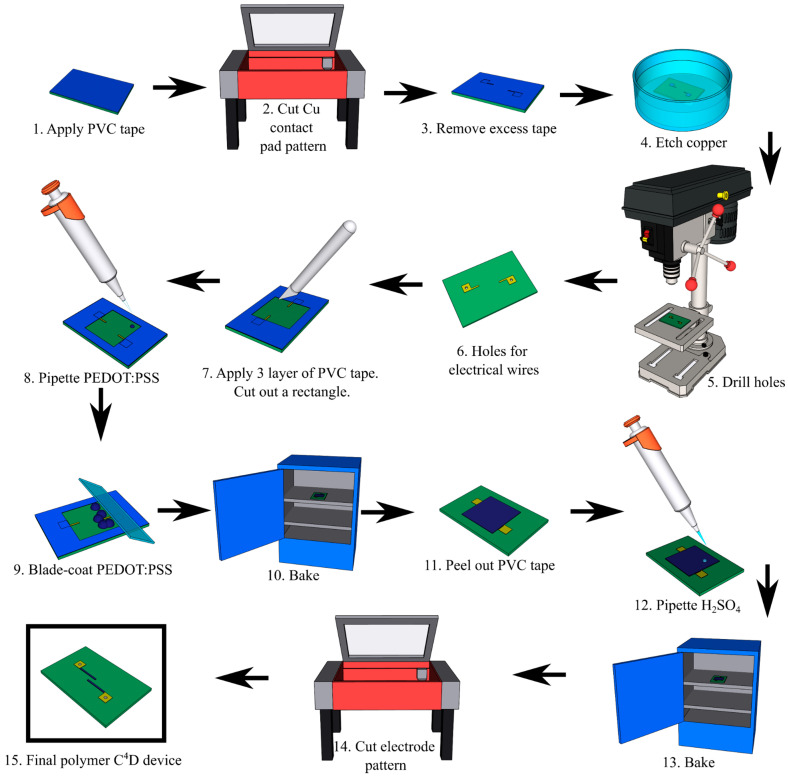
Drop-stain-coat method employed with C^4^D device fabrication procedure. (**1.1**) Apply PVC tape onto FR-4 sheet. (**1.2**) Cut out copper contact pad pattern on the laser cutter. (**1.3**) Remove tape around pads. (**1.4**) Chemically etch FR-4 sheet. (**1.5**, **1.6**) Drill 1 mm holes using benchtop drill press for electrical wire insertion. (**1.7**) Apply 3 layers of PVC tape; cut a rectangular pattern. (**1.8**) Pipette cold-filtered PEDOT:PSS into the rectangular area. (**1.9**) Blade-coat onto the FR-4 sheet using a clean glass slide. (**1.10**) Place in a preheated oven to bake at 120 °C for 20 min. (**1.11**) Cut around perimeter of the PEDOT:PSS film along the edge of the PVC tape with a precision knife and remove all tape layers. (**1.12**) Pipette 6 M H_2_SO_4_ solution onto the plate leaving a small border of untreated PEDOT:PSS around the rectangle. Let rest for 6.5 min. Pipette acid off, wipe excess acid and DI water. (**1.13**) Bake at 120 °C in 30-s intervals until plate is dry. (**1.14**) Raster-cut electrode design into the polymer film on the laser cutter. Remove tape from the sheet. (**1.15**) Solder electrical contacts onto exposed copper contact pads.

PVC tape (McMaster-Carr, Elmhurst, IL, USA) was applied onto copper-clad FR-4 sheet (Figure 1 (1)) and the copper contact pad pattern was cut using a laser cutter (Universal laser systems) (Figure 1 (3)). Only the tape mask was retained; remaining tape was peeled away from the FR-4 sheet (McMaster-Carr, Elmhurst, IL, USA). The sheet was chemically etched by immersing in copper etching solution (Figure 1 (4)) as described in Section 3.1. After ~45 min, the PVC mask was removed and the surface cleaned with 70% isopropyl alcohol (IPA) to remove adhesive residue. A 1 mm diameter hole was drilled into each copper contact pad using a benchtop drill press (Skil, Naperville, IL, USA) (Figure 1 (5)), creating insertion and solder points for connecting wires (Figure 1 (6)). The FR-4 sheet was then covered with 3 layers of PVC tape. A rectangle covering the two copper electrodes was cut using a precision knife. The tape was peeled from the central rectangular region (Figure 1 (7)) and the FR-4 plate was cleaned with IPA. Cold, filtered PEDOT:PSS was pipetted in 200 µL increments into the rectangle (Figure 1 (8)). The droplets were evenly spaced over the rectangle using a clean 1″ × 3″ glass slide (McMaster-Carr, Elmhurst, IL, USA) (Figure 1 (9)). The FR-4 sheet was then placed in a preheated oven to bake at 120 °C for 20 min (Figure 1 (10)). After removing from the oven and cooling, PEDOT:PSS films were cut along the perimeter of the edges of the PVC tape with a precision knife and removed (Figure 1 (11)). The edges of the plate were cleaned with 99% acetone (Sigma Aldrich, St. Louis, MO, USA).

The FR-4 sheet was then treated with 6 M H_2_SO_4_ by pipetting onto the plate in 200 µL increments (Figure 1 (12)), leaving a small border of untreated PEDOT:PSS around the rectangle, then rested for 6.5 min. The acid was pipetted off, and DI water was pipetted onto the plate to cover the area. This was repeated three times. A delicate task wipe (Kimwipes, Sigma Aldrich, St. Louis, MO, USA) was gently laid over the polymer rectangle to absorb any excess DI water. The sheet was baked again at 120 °C in 30 s intervals (Figure 1 (13)) until the plate was dry upon visual inspection.

**Figure 2:**
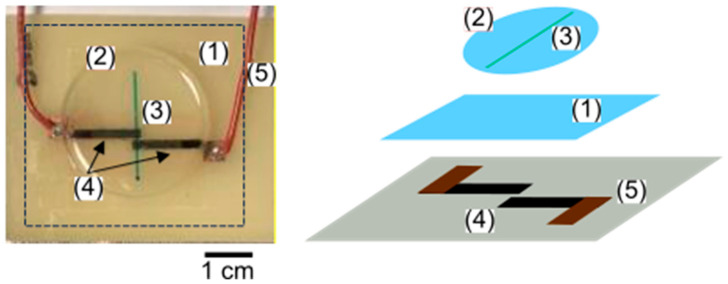
(LEFT) Complete PolyCoDES device. (**1**) PDMS insulating layer to isolate the microchannel and the polymer electrodes. Black dotted line box drawn along the insulating layer edge to guide the eye. (**2**) PDMS microchannel body. (**3**) Microchannel filled with blue dye for better visualization. (**4**) Polymer electrode pair. (**5**) Soldered wires to both electrode copper pads for electrical contact with the rest of the circuitry. Square window in the insulating layer was cut right above the copper pads for soldering. (RIGHT) Exploded view of the insulating layer (**1**), PDMS microchannel body (**2**), microchannel placement in the body (**3**), PCB with polymer electrodes (**4**), and the wires soldered to the copper pads (**5**).

The sides of the sheet were taped off with PVC tape to create a 5–8 mm border on the FR-4 plate ensuring square ends of copper connections were fully covered. The required geometry, 2 mm wide, 0.5 mm apart electrodes were raster cut into the polymer film by laser cutter (Figure 1 (14)). The tape was removed from the sheet. Excess polymer around the electrodes was wiped using micro cotton swabs (Amazon, Seattle, WA, USA) wetted with DI water resulting in the desired electrode geometry (Figure 1 (15)). The prepared polymer films were profiled using a UV laser confocal microscope (Keyence, Japan). Blade-coat PDMS the microchannel PDMS block were prepared as discussed in Govinda Raj et al. [20,21]. The fully fabricated PolyCODES device with the insulating PDMS layer and the microchannel is shown in Figure 2.

### 2.3. C^4^D Hardware Setup and Operation

A bipolar excitation sine wave was generated using a function generator (33220A, Agilent Technologies, Santa Clara, CA, USA) set to 1 Vpp at 800 kHz. The signal was amplified to 10 Vpp using one stage of OPA606KP (Texas Instruments, Dallas, TX, USA) with resulting amplification of 10× and supplied to the excitation polymer electrode. The resulting signal at the sensing polymer electrode was converted to voltage using two stages of OPA606KP, each with an amplification factor of ~28×, resulting in an effective amplification of ~784×. A rectifier circuit was used to output a stable dc signal proportional to the amplitude of the sensing ac signal and was fed into an Arduino NANO (Sommerville, MA, USA) to record data in real-time at 1 datapoint/s on a MacBook Pro laptop. A custom LabVIEW program functioned as a low pass filter with sampling frequency of 150 Hz and low cut-off frequency of 20 Hz. All operational amplifiers were biased at ±15 V using the Rigol bipolar DC power supply (DP831A, Rigol, Cleveland, OH, USA). All wires, resistors, capacitors, and diodes were purchased from Mouser electronics (Mansfield, TX, USA). The excitation signal and the final output signal were monitored in parallel on the digital phosphor o-scope (DPO3012, Tektronix, Beaverton, OR, USA) to detect circuit anomalies, if any. The entire schematic is shown in Supplementary Material SM2.

## 3. Results and Discussion

### 3.1. Device Fabrication Characterization—Electrode Layer Thickness and Resistance

**Figure 3:**
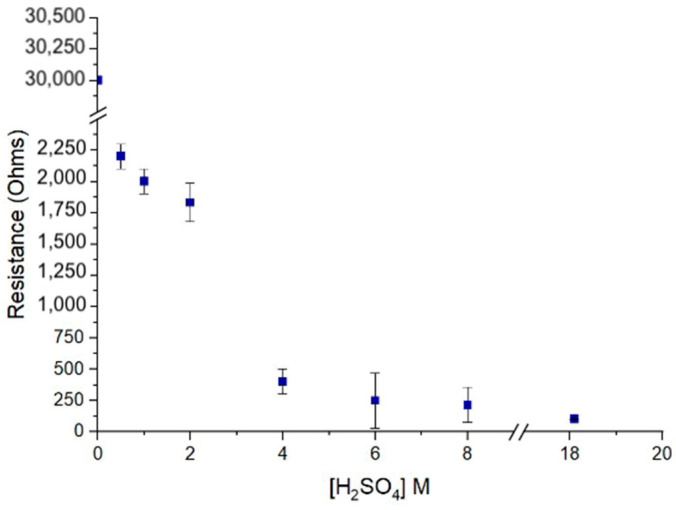
Concentration of H_2_SO_4_ used for polymer treatment versus measured resistance. Acid exposure time was kept constant at 3 min for all concentrations. Error bars represent standard deviation of resistance measurements made at three different spots on polymer films.

The change in conductivity of PEDOT:PSS after treatment with acid is a consequence of the altered ratio of electrically conducting PEDOT to insulating PSS [36], which depends on the concentration of acid and time of acid exposure. To determine lowest concentration required to reduce film resistance, identically fabricated PEDOT:PSS films were subjected to varying sulfuric acid concentrations from 0 to 18.1 M (Figure 3) for 3 min. At 6 M concentration, a resistance of 250 Ω was measured; at higher concentrations, measured resistances did not exhibit a statistically significant change; therefore, 6 M was chosen for further steps to elevate safety concerns.

**Figure 4:**
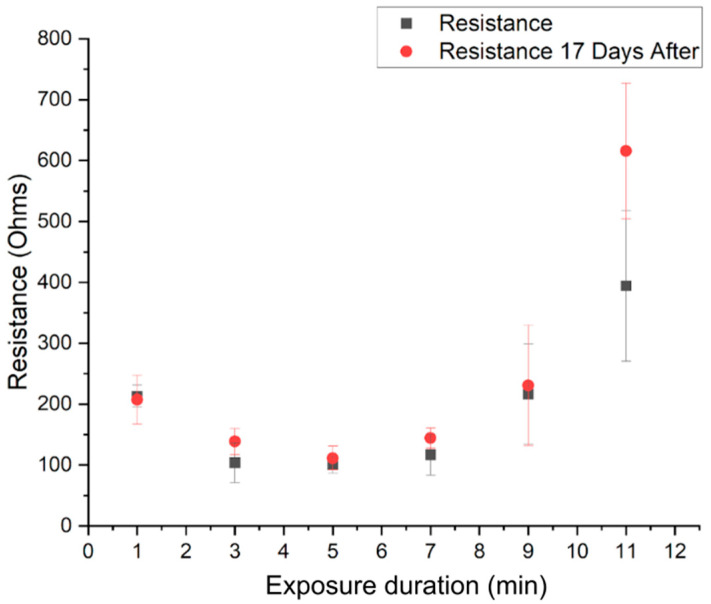
Exposure time of 6 M H_2_SO_4_(aq) versus resistance. Error bars represent standard deviation of resistance measurements made at three different spots on polymer films.

**Figure 5:**
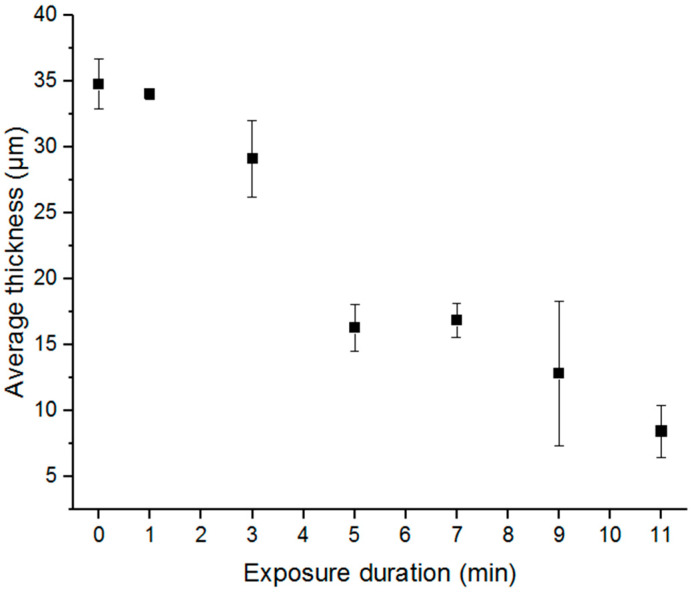
Exposure time of 6 M H_2_SO_4_(aq) versus thickness. Error bars represent standard deviation of resistance measurements made at three different spots on polymer films.

To determine acid exposure time, films were subjected to 6 M sulfuric acid exposure varying in duration from 0 to 11 min (Figure 4). Thicknesses and resistances were measured at three locations spaced 1 cm apart on each film. Thickness measurements were made down from the top surface of the PEDOT:PSS to the top surface of substrate (Figure 5). To minimize computation and processing time, only a 5 mm × 5 mm area of the electrode was imaged for a given trial. With increasing acid exposure time, PSS was progressively removed and resistance decreased, but at longer acid exposure times, PEDOT was also etched, resulting in higher resistance [36], consistent with theory. A 5 min acid exposure was found to yield the most uniform resistance throughout the film. The film retained resistance and uniformity even after 17 days of benchtop storage implying no material deterioration over time.

### 3.2. Excitation Frequency and Amplitude Selection

**Figure 6:**
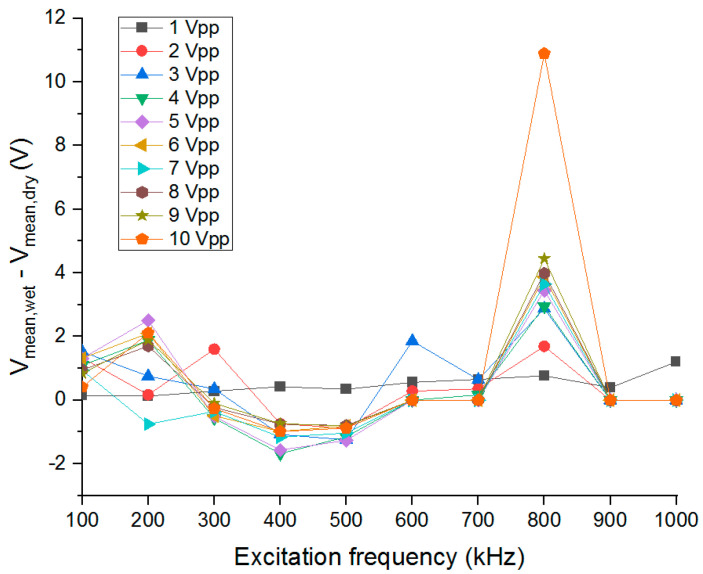
Excitation frequency versus voltage difference between wet and dry channels. The highest V_mean_ difference was measured when the function generator was set to 800 kHz and 10 Vpp, and were therefore chosen excitation signal parameters.

Multiple studies have been aimed at understanding the effect of frequency on C^4^D performance [29,54,55]. Due to varying electronic circuit designs, full effects of frequency are still unknown [29]. Here, stray capacitance of the C^4^D device was empirically determined, finding the highest difference in mean output voltage for dry and wet channels (DI water) was measured with the excitation signal at 800 kHz and 10 Vpp (Figure 6), and were therefore chosen for further study.

### 3.3. Device Fabrication Characterization—Electrode Layout

**Figure 7:**
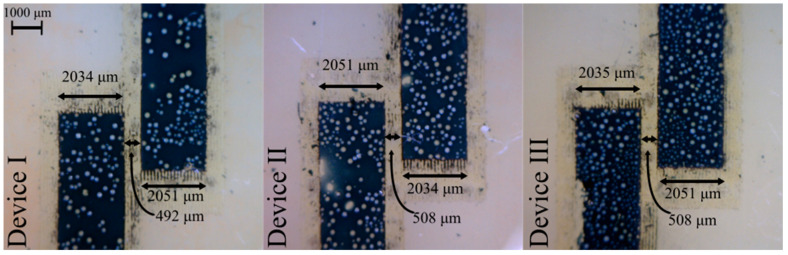
Microscope images of polymer electrodes of three C^4^D devices amplified 10×. Laser-cutter settings were 2 mm-wide electrodes placed 0.5 mm apart.

Typical electrode designs include a grounded Faraday shield to minimize stray capacitance, but with antiparallel orientation and proper electrode geometries, the effect of stray capacitance can be minimized without shielding [29]. Post-fabrication, electrode geometries were measured using a microscope and found to be as shown in Figure 7. The input geometry (software) was 2 mm wide with 0.5 mm separation gap.

Internal laser cutter head auto-positioning margins resulted in wider electrodes yielding smaller separation distance between electrodes, as anticipated. In Device III, a small amount of material of one electrode was removed during fabrication. Since only electrode area directly underneath the microchannel effects output signal, the missing material did not adversely impact device performance.

In Figure 7, all PEDOT electrodes generated were “porous”, and viscous PDMS fluid failed to occupy these micro features during blade-coating procedures, leaving behind numerous micro-bubbles. Bubbles are randomly but statically positioned, adding additional minor dielectric material with permittivity of air. Device performance was not adversely affected; individual device performance was found to be uniform and predictable.

### 3.4. Limit of Detection (LOD) Determination

Three independent 1M stock solutions of four salts were serially diluted to a combination of linearly and logarithmically spaced samples providing twelve data points: 0, 100 nM, 500 nM, 1 μM, 5μM, 10 μM, 100 μM, 500 μM, 1 mM, 5 mM, 10 mM and 100 mM. The linear range (LR) was found to be 1–10 mM for all salts. LOD experiments consistently utilized concentrations between 0 and 8 mM for all salts. New samples within this range were prepared from 1M stock solutions in triplicate to determine LODs (Figure S2 in Supplementary Material SM3). Output voltages were recorded over ~10 s for each salt concentration on the MacBook Pro. During data processing, voltages corresponding to DI water (0 mM) were offset to 0 V in one-step calibration. It was later smoothed using a custom low-pass filter via LabVIEW. LOD was calculated by linear fitting the data, then calculating the concentration at which SNR is ≥3. For each trial, LOD was calculated as shown in Table 1 for each device and each individual salt. ANOVA analyses showed no statistically significant differences in the performance of the three devices (CI = 95%, α = 0.05), which confirmed that fabrication was reproducible, and bubbles did not have a detrimental effect on overall performance of devices.

### 3.5. “Real-World” Sample Testing

Two “real-world” samples were tested on PolyCoDES Device2—ocean water and Rio Tinto water. Ocean water provides an example of relatively high salinity and contains a complex mixture of salts [56]. Rio Tinto is an example of high salinity, extreme acidity, and dissolved heavy metals, containing iron, magnesium, copper, zinc [57] at a pH < 2 [58]. Rio Tinto has an electrical conductivity of ~40 mS/cm (25,600 ppm) [59], converting to 438.06 mM for NaCl and 180.23 mM for Na_2_SO_4_. Ocean water has an electrical conductivity of ~47.5 mS/cm (30,400 ppm) [60], equivalent to 520.2 mM for NaCl and 214.02 mM for Na_2_SO_4_.

**Figure 8:**
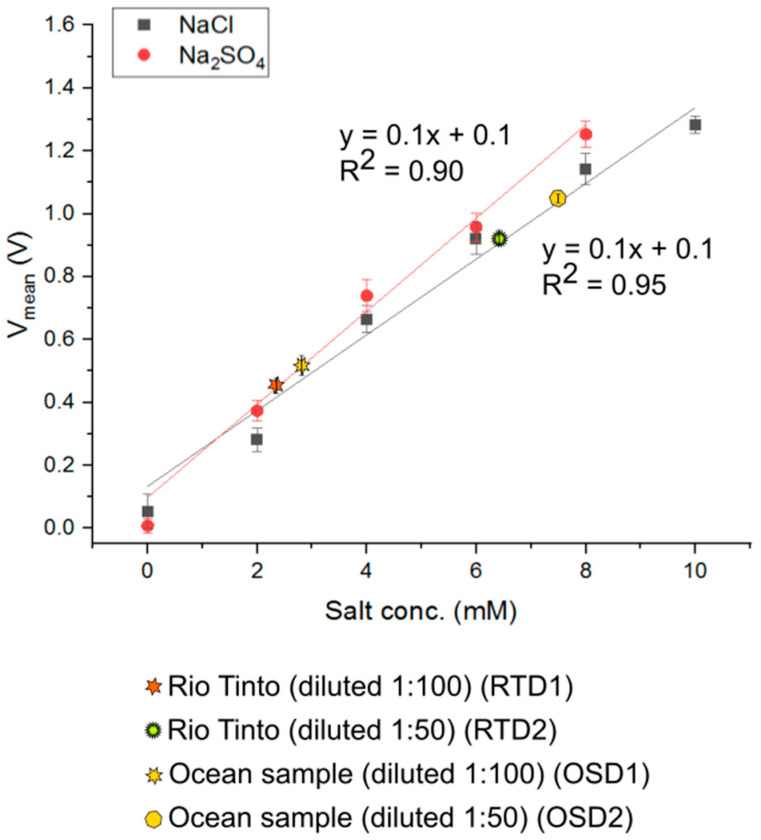
Two “real-world” samples tested on Device II—Rio Tinto water and ocean water. For device validation, they were diluted 1:100 and 1:50. All voltages for real-world samples were plotted against NaCl and Na_2_SO_4_ curves to estimate the total dissolved solids (TDS) content.

For device validation, Rio Tinto and ocean water were diluted 1:100, and 1:50. Sample voltages were plotted against NaCl and Na_2_SO_4_ curves to estimate total dissolved solids (TDS) content. Rio Tinto dilute1 lies on the Na_2_SO_4_ curve at ~2.4 mM and Rio Tinto dilute2 lies on the NaCl curve at ~6.4 mM (Figure 8). This implies that the original TDS content of Rio Tinto is within 240–320 mM. Similarly, Ocean Water dilute1 lies on the Na_2_SO_4_ curve at ~2.9 mM and Ocean Water dilute2 lies on the NaCl curve at ~7.5 mM implying that original TDS content of the sample is 290–375 mM. Both ranges measured are well within the range of conductivity values obtained by commercial probes [59,61] and validate device performance. The samples, dilution ratios, and concentrations are in Table 2. Equations used in calculations are in Supplementary Material SM1.

## 4. Conclusions

PolyCoDES is a low-power, low-mass analytical device employing contactless conductivity detection for quantitation of low concentration dissolved salts. PolyCoDES uses a complete polymer architecture without needing cleanroom facilities—using microfabrication techniques demonstrated in earlier works and integrating drop-stain-coating methods for use C^4^D electrodes. The technique is simple, resource-efficient, and could enable iterative prototyping cycles with high reproducibility, and short turnaround times. Three devices were made with identical fabrication steps and tested with four Europa-relevant salts—NaCl, MgSO_4_, Na_2_SO_4_, and KCl—and validated with two “real-world” samples of geochemical interest terrestrially and considered analogous to extraterrestrial oceans.

ANOVA analyses showed no statistically significant differences in the performances of three independently fabricated devices confirming the fabrication technique’s reproducibility. This is the first report of drop-stain-coating PEDOT:PSS and, to the best of our knowledge, the first demonstration of a polymer C^4^D electrode. At 100s of µM detection limit, this work exceeds NASA LOD requirement of 100 mM [60] by three orders of magnitude.

*In situ* analyses on astrobiologically relevant solar system bodies require access to samples that have been protected from climatic processes, radiation, and space-weathering byproducts on a global scale. Typically, lander missions require costly, bulky, high-energy soft lander platforms that are complex to build. Instrumented penetrator designs housing analytical devices on a microfluidic platform fitting within a low mass, volume, and power consumption envelope have a high potential for enabling efficient, distributed, low cost missions with great quality science return. Penetrator mission instruments need materials that are less susceptible to impact damage to ensure better survivability. Multiple PolyCoDES payloads could ride on a single Discovery-class orbiter mission and be ejected at various points during the orbit to collect samples from geographically spaced locations, thereby enhancing the science return. Robustness to stresses, and reproducibility are critical for space-flight missions, particularly for the ones that employ high acceleration impacts. This work satisfies these key aspects with performance exceeding that requested by NASA in a novel design.

Future work must include circuit optimization with high-speed diodes for fast response to high frequency excitation and temperature correction, which will suitably prepare the configuration for followed by integration with a separation technique like microchip capillary electrophoresis for identification of ionic species in a mixed brine solution. This can be followed by hardware miniaturization to fit it into the sabot dimensions. Impact and flexibility tests are necessary to confirm the physical and functional survivability of the PolyCoDES architecture.

## Figures and Tables

**Table 1:** Lower limit of detection of three devices for Europa-relevant salt analytes.

	NaCl	MgSO_4_	Na_2_SO_4_	KCl
**Device I LOD (mM)**	1.53 ± 0.03	0.14 ± 0.02	1.07 ± 0.04	2.0 ± 0.1
**Device II LOD (mM)**	1.43 ± 0.01	0.31 ± 0.05	1.10 ± 0.08	0.40 ± 0.08
**Device III LOD (mM)**	1.37 ± 0.03	0.17 ± 0.02	1.3 ± 0.1	3.35 ± 0.08
**Linear range (mM)**	0–8	0–5	0–8	0–5

**Table 2:** Two “real-world” samples were tested on PolyCoDES device 2 and plotted against NaCl and Na_2_SO_4_ calibration curves to estimate their total dissolved solids (TDS) content.

Sample	Code	Dilution Ratio	Conc. from Calibration Curves (mM)	Conc. Range Estimate (mM)	Concentrations Reported by Other Groups (mM)
**Rio Tinto water diluted1**	RTD1	1:100	2.4	240–320	180.23–438.06
**Rio Tinto water diluted2**	RTD2	1:50	6.4		
**Ocean water diluted1**	OWD1	1:100	2.9	290–375	214.02–520.2
**Ocean water diluted2**	OWD2	1:50	7.5		

## Data Availability

Data are contained in the article and Supplementary Materials.

## Supplementary Materials

The following supporting information can be downloaded at: https://www.mdpi.com/article/10.3390/s25030775/s1, Figure S1: Circuit schematic diagram of the PEDOT:PSS C^4^D setup; Figure S2: Clibration curves of all four salts on Device III.
