# Supplementary material for: Polymer-Based Contactless Conductivity Detector for Europan Salts (PolyCoDES)
Source: Sensors (Basel). 2025 Jan 27;25(3):775. doi: 10.3390/s25030775 (PMC11820672; doi:10.3390/s25030775)
Supplement: Supplementary file 1 [file sensors-25-00775-s001.zip › sensors-3316721-supplementary.pdf]

Supplementary material

SM1. Equations used for interconversion of TDS, conductivity and molarity

- Commercial TDS meters measure the electrical conductivity of a solution and display the result in PPM using the following formula:

$$ppm_{640} = 640 \times \sigma$$

(Eq. 1)

σ is the solution conductivity in mS/cm

- Molarity was calculated from ppm for each salt species using the following formula:

$$X\ moles/L = ppm_{640}/(molar\ mass \times 1000)$$

(Eq. 2)[53]

SM2. Circuit schematic of the PEDOT:PSS C^4D hardware setup.

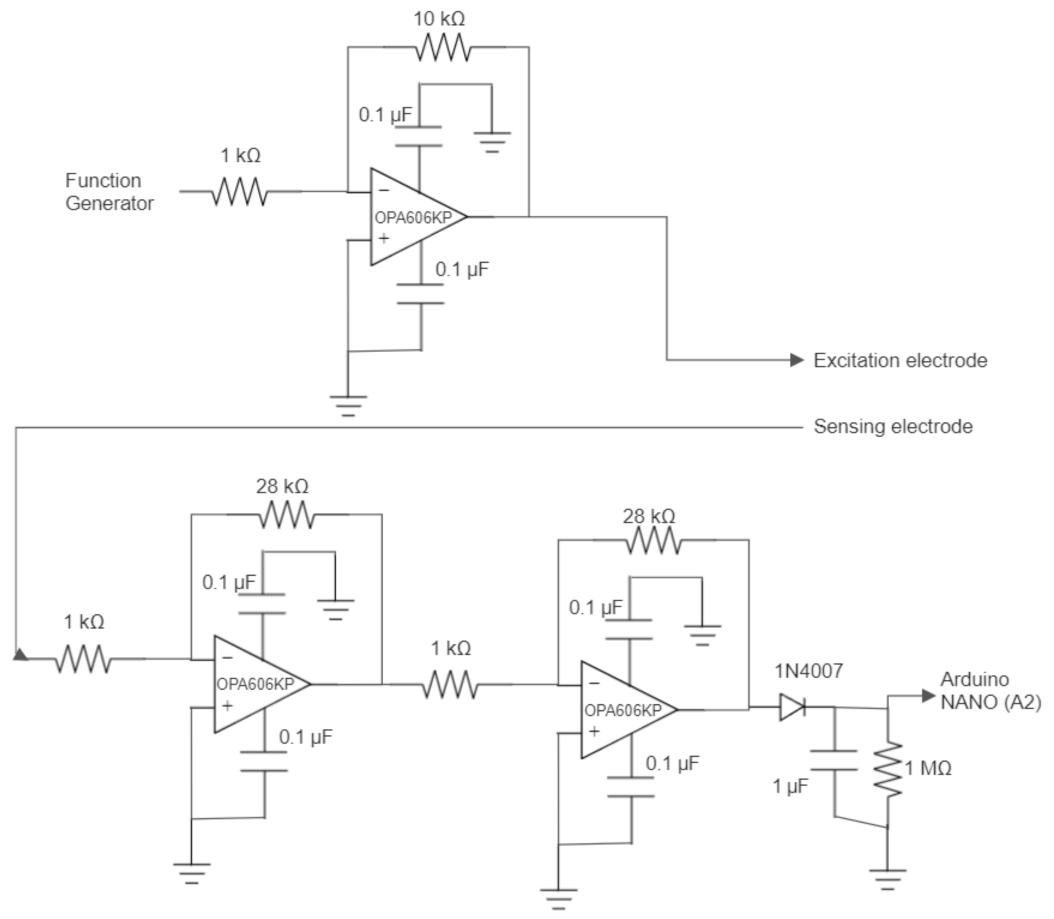

Figure S1: Circuit schematic diagram of the PEDOT:PSS C^4D setup.

SM3. Calibration curves of all four salts as measured on Device III.

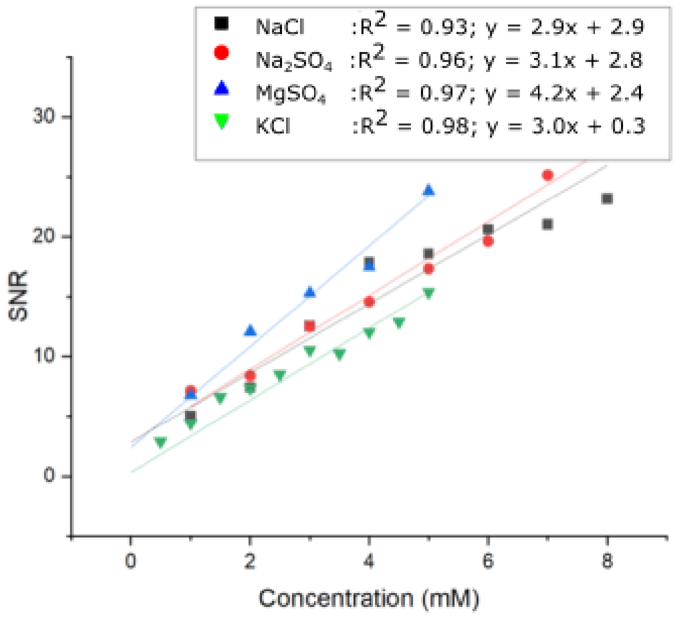

Figure S2: Calibration curves of all four salts on Device III.
